# Evolution of Regulated Transcription

**DOI:** 10.3390/cells9071675

**Published:** 2020-07-12

**Authors:** Oleg V. Bylino, Airat N. Ibragimov, Yulii V. Shidlovskii

**Affiliations:** 1 Laboratory of Gene Expression Regulation in Development, Institute of Gene Biology, Russian Academy of Sciences, 34/5 Vavilov St., 119334 Moscow, Russia; bylino@gmail.com (O.V.B.); dysport@bk.ru (A.N.I.); 2 Center for Precision Genome Editing and Genetic Technologies for Biomedicine, Institute of Gene Biology, Russian Academy of Sciences, 34/5 Vavilov St., 119334 Moscow, Russia; 3 I.M. Sechenov First Moscow State Medical University, 8, bldg. 2 Trubetskaya St., 119048 Moscow, Russia

**Keywords:** enhancer, transcription, evolution, promoter, chromatin loop, gene regulation

## Abstract

The genomes of all organisms abound with various *cis*-regulatory elements, which control gene activity. Transcriptional enhancers are a key group of such elements in eukaryotes and are DNA regions that form physical contacts with gene promoters and precisely orchestrate gene expression programs. Here, we follow gradual evolution of this regulatory system and discuss its features in different organisms. In eubacteria, an enhancer-like element is often a single regulatory element, is usually proximal to the core promoter, and is occupied by one or a few activators. Activation of gene expression in archaea is accompanied by the recruitment of an activator to several enhancer-like sites in the upstream promoter region. In eukaryotes, activation of expression is accompanied by the recruitment of activators to multiple enhancers, which may be distant from the core promoter, and the activators act through coactivators. The role of the general DNA architecture in transcription control increases in evolution. As a whole, it can be seen that enhancers of multicellular eukaryotes evolved from the corresponding prototypic enhancer-like regulatory elements with the gradually increasing genome size of organisms.

## 1. Introduction

Gene expression is a complicated multistep process that requires tight control. Eukaryotes have the corresponding regulatory machinery containing multiple *cis*- and *trans*-elements. The *cis*-regulatory elements are noncoding DNA sequences distributed throughout the genome [1]. Enhancers are one of the most important and best studied groups of regulatory elements. These specific DNA regions act as binding targets for transcription factors (TFs), which form DNA–protein complexes associated with transcription activation. The complexes directly interact with gene promoters and govern their activity in time and space.

The origin of transcription regulation mechanisms of eukaryotes lies in the world of prokaryotes. Therefore, it is not surprising that many features of eukaryotic regulation are common between these two groups of organisms. Thus, it is of great interest to follow evolution of the mechanism whereby transcription is activated step by step. The evolutionary approach obviously allows us to better understand the origin of the transcription regulatory complexity of higher eukaryotes, to clarify the general details of regulation, and to highlight the specific mechanisms and features that are found in the regulation of eukaryotes and are shared with prokaryotes. Here we provide an overview of enhancer-like mechanisms in prokaryotes, describe similarities in these mechanisms shared by both eukaryotes and prokaryotes, and discuss the gradual increase in the complexity of regulation by enhancers in evolution from eubacteria and archaea to unicellular eukaryotes, further to multicellular invertebrates, and finally to vertebrates.

## 2. The Concept of an Enhancer Sequence for Different Organisms

For multicellular eukaryotes, the broadly accepted concept is that a transcriptional enhancer is an independent DNA sequence that is separated from the promoter by a distance in 1D (but not in 3D) and combines sites for positive DNA-binding transcriptional regulators. Classically, a transcription enhancer is understood as a DNA sequence that is able to activate transcription over long distances regardless of its orientation with respect to the core promoter [2]. In this form, the concept of a transcription enhancer is inapplicable to unicellular eukaryotes and surely to prokaryotes.

In unicellular eukaryotes such as yeasts, an upstream regulatory region serving to activate transcription is carefully termed upstream activating sequence (UAS) or sometimes the enhancer-like sequence, meaning that a UAS possesses most, but not all, of the properties characteristic of enhancers of multicellular eukaryotes. UASs cannot activate transcription when located in a gene intron or downstream of the gene in contrast to enhancers of multicellular eukaryotes and are similar to them in being capable of activating transcription when inverted [3,4].

In prokaryotes, the region harboring both binding site for RNA polymerase (RNAP) and binding sites for transcriptional regulators is usually not subdivided into a promoter and bacterial enhancer(s) and is defined as a promoter. However, the term UAS [5,6,7,8], or UAS-like [9], or US region [10] is sometimes used for bacteria. The term is most often used to denote the bacterial enhancer-like sequences involved in an activation loop formation mechanism (see Section 5.4) [11,12,13]. It should be noted that the term UAS is sometimes applied to the promoters of rRNA- and tRNA-coding genes [14,15].

It is clear from the brief explanations above that a united block of regulatory DNA was obviously subdivided during evolution into two independent sections, which were assumed in the recent past to have different functional properties. However, recent works provided evidence that promoters and enhancers of multicellular eukaryotes have much in common and share several properties and functions [16,17,18]. Sometimes, promoters and enhancers are even considered now as a single class of functional elements, with a unified architecture for transcription initiation [19].

Common architecture and functional features (chromatin structure and transcription machinery recruitment) of eukaryotic enhancers and promoters reflect the conversion of one type of elements into the other during evolution of eukaryotes. Indeed, it was found that several hundreds of ancestral enhancers were converted to promoters in primate and rodent lineages of mammals during evolution [20,21].

An interesting hypothesis on the origin of enhancers in eukaryotes has recently appeared to suggest that developmental enhancers of multicellular eukaryotes evolved from unicellular eukaryotic inducible promoters [22]. Here we support this view by showing that, in general, enhancers of multicellular eukaryotes, including developmental ones, evolved via increasing separation of the upstream binding sites of transcriptional regulators from the core promoter as the genome sizes of organisms gradually increased.

## 3. The Origin of Transcriptional Enhancers of Eukaryotes and Evolution of the Genome Size

### 3.1. Principles of Transcription Activation Shared by Both Prokaryotes and Eukaryotes

The architecture of genomes reflects the principles of gene expression regulation. Jacob and Monod’s operon concept [23], coupling signal perception with a genome response in the form of gene activation or repression, proved to be universal for all cellular organisms. In bacteria, the signal perception is directly coupled with an immediate genome response executed by transcriptional regulators, which interface with the general transcription apparatus and couple gene expression with signal transduction.

Two systems were developed by prokaryotes to regulate gene expression in response to stimuli. The first and the simplest one is the so-called one-component system. Transcriptional regulators of these systems are the most abundant and demonstrate remarkable structural similarity, containing both DNA-binding domain and a sensor domain, which can bind small-molecule ligands or metabolites. One-component systems are the most ancient signal-processing systems and are extremely diverse in relation to their function [24].

Two-component systems originate from one-component ones and evolved to respond to environmental stimuli [24]. These systems have more sophisticated structures and process signals from the environment, including metabolites, compounds taken up by the cell, light, temperature, and the redox state. In such systems, histidine kinase is combined in one polypeptide with a membrane-integrated sensor. Once the sensor conformation changes in response to a stimulus, the kinase autophosphorylates and phosphorylates a soluble intracellular transcriptional regulator, which possesses a receiver domain and a DNA-binding domain [25]. After phosphorylation, the transcriptional regulator acquires the property of binding to the target gene to enhance its expression. Many sensory domains are common for one-component regulators and transmembrane histidine kinases [24].

Eukaryotes developed several more systems with complicated cascades of signal transduction from membrane to DNA in addition to the two above signal transduction systems. However, both simple systems of prokaryotes and complex systems of eukaryotes converge on transcriptional regulators or TFs, which can act as activators and repressors. TFs convert incoming internal or environmental information into gene expression output, allowing the cell to adapt to changing conditions.

Promoters act as integrators of different regulative signals provided by transcriptional regulators and are absolutely essential for activation of transcription. Recruitment or driving away RNAP from a promoter is, by and large, a key and unified principle underlying the regulation of transcription from unicellular prokaryotes to vertebrates.

TFs are mostly homodimers in prokaryotes and heterodimers in eukaryotes. Both homo- and heterodimeric TFs occupy distinct and often single sites in a promoter, and each subunit recognizes a DNA sequence of 8–10 base pairs. A couple of sites is known as an operator or a box in prokaryotes. In multicellular eukaryotes, the sites of TFs often occur in many copies over extended DNA regions and are found in enhancers, locus control regions (LCRs), developmental enhancers, and superenhancers and may be tens, hundreds, or even thousands of kilobases away from the transcription start site (TSS), depending on how advanced the organism is evolutionarily.

Another common feature of both simple and complex systems is that binding sites for both activators and repressors may occur together in one regulatory DNA sequence. In prokaryotes, a good example is provided by the system of the *lac* operon. The *lacZ* promoter harbors two binding sites for a Lac repressor, which is a product of the *lacI* gene and acts to prevent *lacZ* transcription in the absence of lactose. In addition to LacI binding site, the *lacZ* promoter has one binding site for the transcriptional activator known as CAP or CRP (catabolite activator protein or cAMP receptor protein), which acts to stimulate *lacZ* transcription at lower glucose concentrations [26]. Complex systems of multicellular eukaryotes, for example, embryonic enhancers (initiators) of the *HOX* genes, harbor many sites for different repressors and activators [27]. Embryonic enhancers respond to morphogenetic gradients of maternally loaded proteins and other regulators, and the balance of sites for repressors and activators determines whether or not the gene is expressed in a particular body segment [27].

### 3.2. Separation of Enhancers from the Promoter with an Increase in Genome Size during Evolution

Binding sites for regulatory proteins evolved to be farther away from the TSS as genomes grew in size and complexity. For example, the genome of *Escherichia coli* consists of 4.6 Mb of DNA, and the binding sites for the PhoB protein, the master regulator of the Pho regulon, which is responsible for phosphorus assimilation in bacteria, are in the immediate vicinity of the −10 and −35 core promoter elements or even overlap them [28]. A direct physical contact can thus be established between the PhoB regulator and the RNAP–σ^70^ complex [29].

The unicellular eukaryote *Saccharomyces cerevisiae* has a 12.1-Mb genome, and the binding sites of regulators in the enhancer-like UAS [3] are usually several hundreds of base pairs away from the core promoter, at maximum 1 Kb [30,31]. In this case, the interaction of the activator with general TFs and RNAP requires a loop formation between the enhancer and promoter [32] and the Mediator complex, a crucial eukaryotic multi-component transcriptional coactivator [33]. An increase in the distance between the UAS and the core promoter results in loss of function [34]. The observation provided a basis for designing an elegant reporter system, which makes it possible to monitor long-distance interactions between regulatory elements and proteins in the genomes of higher eukaryotes; its efficiency was well demonstrated with transgenes [32,35].

The majority of known enhancers are within 10 kb of the promoter in invertebrates, such as *Drosophila*, whose genome is 175 Mb and is about 20 times smaller than the human genome. At the same time, in this organism, up to 20% of all enhancers are long-range enhancers, which may jump over several genes to reach their target promoters [36,37]. The mean number of enhancers interacting with one promoter was estimated at five per promoter in *Drosophila*, like in humans [1,38]. The *svb* and *cut Drosophila* genes provide well-characterized examples of the loci where long-distance interactions between enhancers and promoters are involved in the regulation. Each of the genes has at least five proven enhancers, the farthest of which are approximately 80 kb away from the TSS [39,40]. This redundancy of transcriptional enhancers confers phenotypic robustness because the transcriptional output is guaranteed even in the case of a mutation or accidental loss of one enhancer [39]. Another strong long-range (235 kb) interaction in *Drosophila* was found between the regulatory regions of *scyl* and *chrb* [36]. An extreme example of regulatory complexity in terms of spatial interactions is provided by the *Drosophila* BX-C locus, which is approximately 320 kb in total size and regulates the development of the posterior half of the fly body. Both short- and long-range enhancer–promoter interactions were detected in this locus. These interactions are often mediated by boundary elements and insulators, which are located between BX-C domains [41]. An additional complexity level is added to the BX-C by Polycomb response elements (PREs), which are dispersed throughout BX-C and are associated with epigenetic memory and silencing of genes by forming spatially independent domains [42,43,44]. However, such regulatory complexity is an exception rather than a rule in *Drosophila*.

Long-range interactions reach a huge scale in vertebrates and, in particular, in humans, whose genome is 3300 Mb. For example, the human ZRS enhancer acts over 850 kb to regulate the *Shh* promoter [45], and enhancers of the SOX9 gene are 900 and 1300 Kb away from its promoter [46]. In addition to classical enhancer–promoter interactions, promoters acting as enhancers [47,48,49,50] and extensive enhancer–enhancer and promoter–promoter interactions [51] were detected in human, reflecting the complexity that is characteristic of the regulation in higher eukaryotes and is associated with complex 3D chromatin organization of the genome.

Thus, mammals have the largest genomes, the most complex regulation, and the most distant enhancers among all organisms. The estimated number of enhancers is up to ten actual enhancers per one gene in mammals [1].

### 3.3. Distancing of Enhancers from the Core Promoter in Evolution

While the distances between regulatory regions obviously grew over the course of evolution, it is not quite clear if this change provides an advantage to the organism. Moreover, an increase in distance between *cis* regulatory elements requires the development of special complex mechanisms that ensure the convergence of these elements in space (discussed in part 9). From this point of view, one could speculate that the increase might be a consequence of Kimura’s neutral evolution rather than adaptive evolution [52]. The appearance of additional DNA between enhancers and promoters and the increase in distance between them might arise for the same reasons as the appearance and increase in size of introns in evolution of eukaryotes and may be associated with a decrease in effective population size as organisms gradually became more complex [53]. The insertion of mobile elements serving as sites for homologous recombination during unequal crossing over might be another factor that increased the distance between *cis* regulatory elements in evolution, leading to a duplication of the DNA between *cis* regulatory elements [54].

On the other hand, one could speculate that a bacterial promoter-proximal upstream regulatory region is a tool with a very limited regulating capacity. Gene expression requires much more complicated control in Metazoa [55]. A separate location of enhancers provides evolutionary plasticity to the regulation, for example, allows the situations where one gene is regulated by multiple enhancers or, vice versa, expression of different genes is regulated by one enhancer.

## 4. Principles of Transcription Activation in Eubacteria

The origin of transcription regulation is in the prokaryotic world. In order to see whether parallels could be drawn between enhancer-like mechanisms found in prokaryotes and in higher eukaryotes, it is required first to consider the general principles of transcription activation in eubacteria.

### 4.1. The Role of the Promoter Structure

Bacterial promoters have a modular structure the same as that of eukaryotic promoters and appear to have evolved by a pick-and-mix mechanism, which produced the hierarchies of promoter activities that permit a 1000-fold dynamic range in transcript initiation frequencies [56,57,58]. A promoter may be activator dependent because it carries one or more “imperfect” elements so that the initial RNAP binding is reduced. Furthermore, some promoters have so non-consensus sequences that they exhibit no activator-independent activity. The purpose of activators in all these cases is to compensate for the weak or missing core promoter elements by mediating the interaction between DNA and the σ and/or α subunits of RNAP. Conversely, when all core promoter elements required for transcription are present in the promoter sequence (the so-called strong constitutive promoters, i.e., P_tac_ [59]), the initiation of transcription may occur without activators. Thus, activator-dependent promoters in bacteria are simply the promoters that have weak non-consensus core promoter sequences, in which activator–weak promoter–RNAP interactions replace strong promoter–RNAP interactions.

Bacteria use two distinct sets of mechanisms for transcription activation focusing either on the promoter or on RNAP. In RNAP-centric mechanisms, some factors interact with RNAP to alter its promoter preference. For example, alternative σ factors do this [60,61]. In other cases, the RNAP–σ^70^ interaction is altered by activators that redirect the complex to a certain class of promoters, the process being known as the RNAP appropriation [62].

In promoter-centric mechanisms, activators interact with the promoter to improve the ability of RNAP to initiate transcription, either replacing missing core promoter determinants or blocking the action of a repressor, e.g., by competing for the same binding site. Two common ways are used in bacteria to activate transcription. One is related to the interaction of the C-terminal domain (CTD) of the α subunit of RNAP with the UP element located upstream of the core promoter. The second one involves the interaction of the σ4 domain of σ^70^ with the −35 element. An activator interacts with the σ4 domain and promotes its binding to the “non-perfect” −35 hexamer. Only one of the two subunits of the activator on DNA makes contact with the σ4 domain of σ^70^ upon activation because RNAP carries only one σ factor. To make contact with σ4, activators have to be precisely positioned on DNA and bind to the same face of the DNA helix as the σ4 domain does. The binding of an activator to a site adjacent to or overlapping the upstream end of the −35 hexamer is often triggered by phosphorylation of a receiver domain of the activator or by binding to an activatory ligand, for example, a sugar. This is the case with the AraC regulator at the P*_BAD_* promoter when arabinose binding changes the AraC properties and converts AraC from a repressor to an activator (see Section 5.2 and [63]).

### 4.2. The Role of the Promoter Conformation

One more fundamental form of regulation is based on changing the DNA topology and is widespread from bacteria to human. In bacteria, activation of expression by changing the promoter conformation is a special case of this regulation. Experiments with synthetic promoters that form an angle of 90° in vitro showed that this sharp DNA bend may facilitate the binding of upstream DNA to the backside of RNAP [64]. In vivo, some activators alter the conformation of the promoter DNA to bring distant DNA regions to each other. For instance, target promoters of MerR and other related activators are defective because of the nonoptimal spacing between the −35 and −10 elements [65]. In the situation where RNAP initially binds to the UP element and −35 element, the −10 element is misplaced and promoter melting, unwinding, and interaction of the −10 element with the σ2 domain of the σ^70^ subunit are hindered. This hindrance is overcome by the activator MerR, which causes a localized distortion to align the −10 element with the −35 element and thus triggers transcription activation [66].

Binding of one activator to DNA and subsequent DNA bending may facilitate a direct interaction of another activator with RNAP. For example, the CAP activator-induced bending was shown to modulate the location of binding of the activator MalT and to trigger transcription activation from the malK promoter [67].

Altering the promoter conformation in bacteria may involve nucleoid-associated proteins (NAPs), which are evolutionary predecessors of eukaryotic histones. Their binding to a distal promoter results in translocation of superhelical energy from upstream binding sites (supercoiling-induced DNA duplex destabilized region) to the promoter-proximal region, which is unwound within the open complex, and may change the promoter activity by several dozens of times [8,15,68,69].

NAPs, H-NS, and FIS may interplay with special shape-sensitive activators containing winged helix-turn-helix (wHTH) motifs. wHTHs use an alpha helix to read the base sequence in the major groove while inserting a beta sheet «wing» into the adjacent minor groove [70,71]. wHTH binding to relaxed DNA appears to generate a locally supercoiled state, which assists promoter activation by relocating supercoiling destabilization of DNA strands.

## 5. Enhancer-Like Mechanisms in Eubacteria

In this part we provide specific examples related to bacterial UAS-like regulation. These include direct stimulation of the RNAP complex by an activator, activation loops, repression loops, activator tracing/scanning from a landing enhancer-like site to a core promoter, and rings that mediate interactions between distant DNA regions.

### 5.1. Stimulation of RNAP by an Adjacent Activator

**Figure 1:**
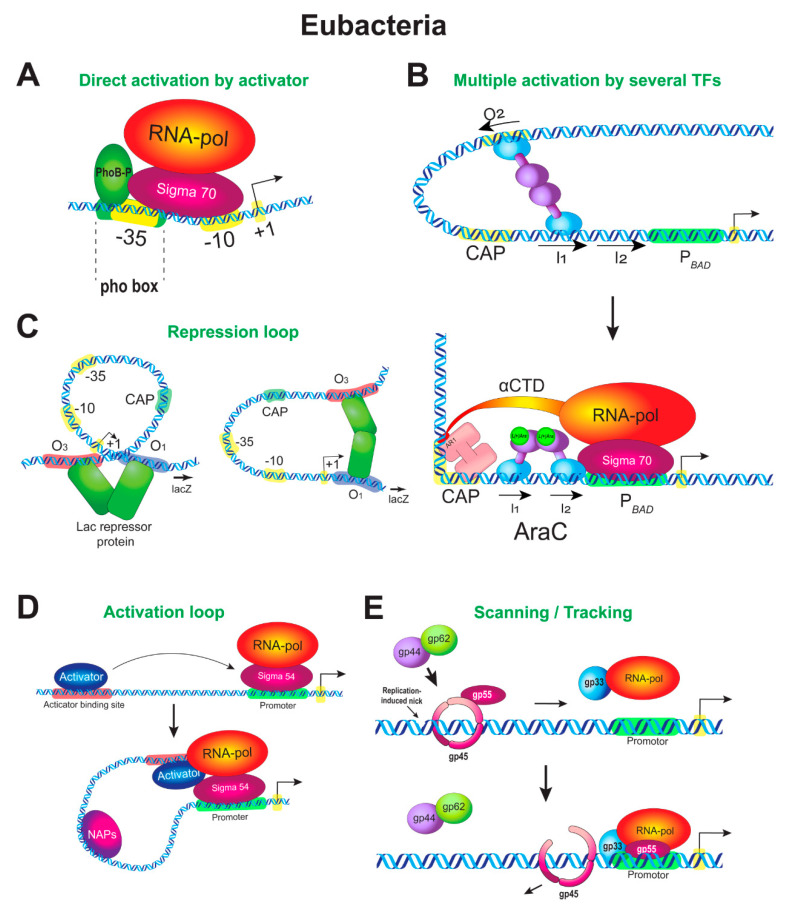
Enhancer-like mechanisms of transcription regulation in prokaryotes. (**A**) Stimulation of RNAP by an adjacent activator. Pho-regulon genes are responsible for adaptation of bacteria to phosphorus starvation. Expression of the Pho-regulon genes is regulated by a two-component system through direct contact of an activator PhoB with the RNAP–σ^70^ complex. Lack of phosphorus in the medium leads to phosphorylation of PhoB (PhoB-P), which then acts as an intracellular soluble response regulator. PhoB-P as a dimer binds to its binding sites at the promoters of Pho-regulon genes (pho-boxes), thus compensating for the weak −35 element, and recruits the RNAP–σ^70^ complex to the promoters. The physical interaction of PhoB-P with σ^70^ in the region of the −35 element is shown. (**B**) Multiple activation of RNAP by several activators. Regulation of the *araBAD* promoter is shown. The upper panel illustrates the repression state in which the promoter is repressed by the AraC regulator. The lower panel shows the transition from repression to activation, which occurs by changing the conformation of the promoter DNA. The repression loop is broken by the CAP protein; the promoter is bent by CAP and the site of *AraC* binding is switched. Changing the promoter DNA conformation allows the activators to interact with RNAP. (**C**) Repression loop. Two alternative conformations of the *lac* operon promoter are shown. The lac-operon is regulated by a one-component regulatory system. The system includes the Lac repressor protein (LacI), which acts as both a lactose sensor and a transcriptional regulator. In the absence of lactose (or in the presence of glucose), the Lac repressor binds to several operator sites at the P_lac_ and negatively regulates expression of the *lacZYA* operon through the formation of a repression loop between the operator sites. The binding of lactose or IPTG affects the LacI conformation, causing LacI to disconnect from the operator sites, and the repression loop disappears. (**D**) Activation loop. An activation loop forms when *glnALG* operon expression is upregulated by the NtrC regulator. When ammonia is depleted in the medium, NtrC undergoes phosphorylation, assembles into heptamers, and binds to an upstream enhancer-like sequence at the promoter of the *glnALG* operon. The loop forms between the NtrC binding sites and core promoter. NtrC remodels the RNAP–σ^54^ holoenzyme into the open complex. The remodeling reaction is ATP dependent. Nucleoid-associated proteins (NAPs) may facilitate the looping mechanism by bending the DNA between the enhancer and promoter. (**E**) Activation ring. The scanning/tracking mechanism of regulation of bacteriophage T4 late genes is shown. A component ring consisting of three gp45 phage polypeptides is put onto DNA at the enhancer region by the loader complex gp44–gp62. A part of the split σ-subunit known as the gp55 protein (σ55) tracks along DNA as a gp45 ligand. At the gene promoter region, the ring activation complex encounters RNAP with gp33 (a second part of the split σ) and mediates the transition of the closed complex to the open one and activation of transcription.

The simplest mode of the activator function is when an activator compensates for the weak core promoter sequence directly, by interacting simultaneously with RNAP and a promoter and thus increasing the frequency of the appearance of the initial RNAP–promoter complex. An example of the direct interaction of an activator with the RNAP–σ^70^ complex is provided by inducible genes of the Pho regulon, which is involved in the cell response to deprivation of phosphorus (Figure 1A). The Pho-regulon genes are regulated by the activator PhoB and usually have weak –35 elements; PhoB is required to stably recruit the RNAP–σ^70^ complex to such promoters. Pho-boxes, on which PhoB is bound, are located upstream of the core promoter or overlap the –35 element. PhoB is phosphorylated upon induction and binds to the Pho-box as a dimer. RNAP is not recruited to the promoter in the absence of the activator [72].

As evident from structural studies, the interaction of PhoB with σ^70^ causes a reorientation of the β-flap tip helix and σ4 domain of σ^70^ in the surroundings of the nascent RNA exit channel, suggesting that the remodeling of RNAP might alter its opening to facilitate the exit of the mRNA, thus reducing the probability of abortive transcription [29,73]. The activation of this type is highly similar to a eukaryotic system known for housekeeping genes, where activator sites are close to the core promoter and where fast RNAP turnover takes place.

Another well-studied example of direct interactions with RNAP is a mechanism whereby the λ cI repressor activates expression of its own gene from the native λ P*_RM_* promoter [74]. The activation by the cI protein also involves the σ4 domain of σ^70^. In this case, not accompanied by any structural change in any protein participant, the cI repressor and σ^70^ are immediately adjacent to the DNA and interaction proceeds through small clusters of amino acid side chains.

### 5.2. Multiple Activation of RNAP by Several Activators

This mechanism is observed for complex promoters, which combine DNA binding sites for several different regulators. At least two regulators act in the simplest case. The first one is an activator, and the second one is of dual nature, acting as an activator or a repressor depending on the situation.

The *araBAD* promoter provides an example of such complex regulation. This promoter is regulated by two TFs, the activator CAP and AraC, which is of dual nature. P*_BAD_* has two direct-repeat half-sites, I1 and I2 (8 bp each), for the AraC activator-repressor. The half-sites are centered at position −52.5 bp (five turns of the double helix in a B shape, of 10.5 bp each) and partly overlap the −35 region. One more AraC half-site, O2, is 211 bp upstream (−265 to −294 from TSS). When arabinose is absent, the subunits of the AraC interact with two nonadjacent DNA sites and a repression loop forms between them [75]; i.e., AraC behaves as a repressor in this case. In the presence of arabinose, one AraC subunit disconnects from O2 and binds to the I2 half-site, which is adjacent to the I1 half-site. This is accompanied by the loss of the repression loop, and AraC gains the ability to interact with RNAP; i.e., AraC behaves as an activator in this case (Figure 1B).

Thus, the reorientation of one subunit of AraC to another site regulates the DNA looping in P*_BAD_* and specifies the activator–RNAP contacts. This mechanism reminds the mechanism of loop formation by a cohesion complex in eukaryotes, when the orientation of two CTCF protein binding sites specifies the direction of loop formation. Changing the polarity of one CTCF site compromises enhancer–promoter interactions [76,77,78,79]. Moreover, switching the interaction from one CTCF site to another can specify regulatory effects in eukaryotes, like in the case of P*_BAD_* [80,81].

The site for the other regulator of P*_BAD_*, the activator CAP, is upstream of the AraC site and is centered at position −93.5. Although there is no detectable interaction between AraC and CAP upon their binding to DNA [82], experiments in vitro showed that CAP facilitates the breaking of the DNA loop if situated on the same face of DNA no more than 4 helical turns upstream of the loop-formation site [83]. CAP does not significantly activate P*_BAD_* when acting alone, in the absence of AraC [84]. Conversely, although AraC binding to both I1 and I2 sites in the absence of CRP is sufficient to stimulate the open complex formation, CAP is required for maximal promoter activity [84,85] and causes an eightfold increase in the rate of RNAP open complex formation on wild-type P*_BAD_* in vitro [85]. Thus, CAP functions as a typical eukaryotic coactivator on P*_BAD_*, enhancing the action of AraC, but is capable of binding to DNA unlike eukaryotic coactivators.

What mechanistic consequences arise when two different activators interact at once with RNAP upon activation? One scenario is that each activator induces its own specific rearrangement of RNAP and each of these rearrangements contributes to the final organization of the complex and transcription output. Another scenario is that both activators do the same job, that is, each of them repeats the other. Experiments testify in favor of the latter; i.e., both CAP and AraC stimulate the recruitment of RNAP as well as the transition to the open complex [63].

Upon activation, the CAP–AraC–RNAP complex substantially bends the DNA, and it is very likely that CAP is positioned close to RNAP despite the fact that the AraC binding site lies between the binding sites for CAP and RNAP (Figure 1B). One α subunit of RNAP utilizes its C-terminal domain to contact the AR1 (activation region) of the closer CAP subunit, and the σ4 domain of σ^70^ presumably contacts the AR3 region of CAP [84]. Both AraC and σ^70^ interact with the −35 element, but AraC interacts with σ^70^ very slightly [86]. In contrast, it was shown in vitro that AraC binds RNAP very tightly [87].

Thus, the example above demonstrates that bacteria already display complex interplay of different regulators on the enhancer-promoter region.

### 5.3. Repression by Looping Prior to Activation

Regulation of gene expression by looping is common in eukaryotes during development [88,89,90], but specific molecular details of these mechanisms have just begun to appear. In this section we give examples of repression loop formation in prokaryotes to illustrate in detail how the repression can be replaced by activation and how this can be related to the DNA topology.

Repression loop formation was studied in bacteria in detail using several examples. The mechanism of how repression loops form in the *araBAD* promoter was described in Section 5.2 (Figure 1B). The loop formation between the repressor sites was confirmed for P*_BAD_* using microscopic [91] and biochemical methods [75,92]. Repression was impaired when the operator sites are on different sides of the DNA helix, emphasizing a role of the proper DNA topology in repression loop formation [93,94]. It was shown that repression loops of up to 500 bp can form using AraC [94].

Using microscopy, repression loop formation was also demonstrated for the *lac* promoter [95] (Figure 1C) and the bacteriophage λ cI-repressor-operator system [96,97]. The findings confirmed that this way of regulation is universal for cell and viral genes. In the case of P*_lac_*, when loops between operator sites are stabilized by the Lac repressor, RNAP appears not to be trapped or occluded in these loops [98]. The mechanism of repression probably consists in that RNAP cannot initiate from the tightly bent promoter because the enzyme lacks sufficient binding energy to untwist the constrained DNA within the loop [98]. The formation of a DNA loop was favored by correct phasing of the two lac operators on DNA [95,96,97].

Repression loops are stabilized in the presence of NAPs, which are functional evolutionary ancestors of eukaryotic histones and HMG proteins, and are destabilized by NAP deletions [99,100,101]. All of these examples have a common feature: in the case of repression, the loops are relatively small, no longer than several hundreds of base pairs [102].

Thus, the formation of a repression loop between DNA-binding transcriptional regulators associated with the correct DNA topology is a universal property of both prokaryotes and eukaryotes.

### 5.4. Activation Loops

An example opposite to the above two ones concerns the functioning of the so-called bacterial enhancer-binding proteins (bEBPs). Activators of this class bind remotely upstream of the TSS and induce the formation of large activation loops. bEBPs are special hexameric ATPases that use ATP hydrolysis to remodel theRNAP–σ^54^ complex [103]. In addition, it was shown that bEBP binding sites can isolate or filter readthrough transcription from upstream genes [13,104]. This property makes these sequences similar to eukaryotic insulators.

There are two major families of σ factors in bacteria, based on sequence homology and the mechanism of action: σ^70^ and σ^54^. Upon binding to the promoter, the RNAP–σ^70^ holoenzyme forms a closed complex, which can spontaneously isomerize to form an open promoter complex [60,61]. Due to this property, σ^70^ is involved in the bulk of cell transcription during active growth, including all essential genes in *E. coli*. Unlike σ^70^, σ^54^ directs transcription of tightly regulated genes involved in the cell response to stress, including nitrogen and carbon starvation, antibiotics, and loss of membrane integrity [95]. σ^54^ binds to RNAP to form a stable closed complex, which rarely spontaneously converts to the open one [105,106]. bEBPs facilitate the transition (isomerization) of the closed complex to the open one using ATP hydrolysis [107], resulting in further DNA melting and loading of the template strand of DNA into the RNAP active site [108,109]. Energy is transferred to the closed complex through a physical interaction between σ^54^ and the AAA+ activation domain (AD) of the bEBP [110,111]. This is functionally analogous to enhancer-dependent initiation of eukaryotic RNAP, which requires an input of energy from ATP hydrolysis provided by TFIIH [112,113].

The best-studied example of activation loop formation by bEBPs is provided by the system of the *glnALG* operon (Figure 1D). When nitrogen is in excess, the nitrogen regulator I (NRI) NtrC exists in a non-phosphorylated form as a dimer and only weakly binds to a pair of its tandem sites in the upstream promoter of *glnA*. When nitrogen is in deficiency, NtrC is phosphorylated by NRII membrane kinase and occupies its binding sites, ~90 and ~140 bp upstream of the *glnA* TSS. The phosphorylated NtrC dimer forms heptameric rings and uses the energy of ATP hydrolysis to physically remodel the RNAP–σ^54^ (NtrA) complex. Interestingly, the distance from the NtrC binding site to the TSS should be no less than 70 bp to allow activation. The distance can be increased up to 1000 bp or more without impairing the activation appreciably [114]. Recently, activation was found to occur even when the distance is 6000 bp, although being less efficient [115].

Electron microscopy demonstrated the presence of looped DNA *in cis*, with the length of the loop being consistent with the spacing between the NtrC binding sites and the core promoter. Once the closed complex is converted to the open one, the interaction of a bacterial enhancer with the promoter is terminated, the DNA loop is opened, and σ^54^ dissociates after promoter clearance by RNAP [116,117].

Studies with a system of two plasmids in which one plasmid carried the promoter and the other one had activator binding sites showed that activation is possible not only *in cis*, but also *in trans*, and that the *in trans* interaction was enhanced with the increasing NRI concentration [117]. A similar concentration-dependent effect was detected in experiments with the native locus, where NRI was shown to be capable of activating transcription even in the absence of an upstream DNA binding site when its concentration is four- to fivefold higher than that required in the presence of the upstream site [118]. These experiments indicate that loop formation per se is not essential for NRI-dependent activation and that NRI binding sites near the promoter are necessary for increasing the local concentration of the activator in the vicinity of the promoter.

The most interesting finding is that the closed RNAP–σ^54^ complex occurs on the promoter of the *glnALG* operon even in the absence of nitrogen deficiency and in the absence of the NRI activator [119]. This feature makes the *glnALG* operon similar to the eukaryotic genes that are regulated by stalling RNAP and are constitutively associated with the RNAP–TFIID complex even when these genes are inactive [120].

The relationship between activation and helical phasing was demonstrated in the case of the activation loop as well, although not for the *glnALG* operon. Binding sites of the NRI activator are in the DNA major groove on one side of the DNA helix, while σ^54^ binding sites are in the major groove on the other side of the DNA helix in the *glnALG* promoter [119]. This is in contrast to other regulation systems described above (lambda repressor, lac repressor, AraC), in which two remote protein binding sites appear to lie on the same helical face when functioning optimally. A proper helical facing might be less relevant in the case of the *glnALG* promoter because the promoter has three activator binding sites and monomers bound at each site possess flexible activation domains pointing in opposite directions [119]. This makes activation systems based on NtrC very flexible. At the same time, a σ^54^–NRI-regulated system with two NtrC binding sites was indeed face-of-the-helix dependent in another organism [121]. Thus, particular promoter properties may be determined by the number of bEBP binding sites available.

How frequent may such type of eukaryotic-like regulation be in prokaryotes? σ^54^ is present in an estimated 60% of bacterial genomes [122]. In *E.coli*, σ^54^ was experimentally shown to regulate 135 of 4 288 protein-coding genes [123]. A number of bEBPs have been characterized and many σ^54^-dependent promoters have been predicted to date [103]. These promoters have bEBP-binding sites up to 3 kb upstream and 1.5 kb downstream of the TSS. Some of them were tested experimentally, and loop-dependent activation was verified. It was found that the looping mechanism at these promoters is facilitated by NAPs that bind between the enhancer and promoter and bend DNA [102,124].

Thus, the activation loop mechanism in bacteria is closest to what we expect from eukaryotic enhancer-dependent regulation. At the same time, the formation of an activation loop is not the only mechanism that spatially associates distant DNA regions in prokaryotes.

### 5.5. Activation Rings

Ring complexes that encompass the DNA molecule and slide along it are widely used in bacteria and eukaryotes (PCNA, cohesin-condensin, etc.). The role of a sliding ring in regulating gene expression by a scanning/tracking mechanism was discovered and studied on late genes of the bacteriophage T4. A sliding clamp comprised of a trimer of gp45 polypeptides is loaded to a distal enhancer-like site by the gp44–gp62 clamp loader complex (Figure 1E). This site can be upstream or downstream of a gene. The enhancer site is not a specific nucleotide sequence in this case, but a nick introduced in the non-transcribed strand and associated with normal DNA replication or recombination. The activation ring slides from the loading site to the promoter of the gene, carrying a part of the split σ-subunit as a ligand (gp55 protein or σ55, a functional analog of the σ2 domain of σ^70^) [125]. On the gene promoter, the activation ring binds RNAP with the gp33 protein (another part of the split σ-subunit) attached to RNAP. gp33 binds to the so-called β-flap, a flexible tip of the β subunit of RNAP [126]. The β-flap normally binds to the σ4 domain of σ^70^, and the σ4 domain recognizes the -35 element. Thus, gp33 mimics the σ4 domain of σ^70^. The promoters of late T4 genes have a −10 element only. Thus, a blocking of the σ^70^-subunit binding site on RNAP by gp33 allows transcription to be activated from late T4 promoters lacking the –35 element. Interactions of the gp45 trimer with RNAP allows the transition of the σ55 (gp55)-RNAP complex from the closed state to the open one and activation of transcription [127].

### 5.6. Rings Mediating Convergence of Distal DNA Sites

Condensins of prokaryotes and eukaryotes and cohesins of eukaryotes are collectively called SMC proteins and are evolutionarily related conserved multi-subunit complexes that form rings of various structures. Condensins/cohesins are vitally important for cell division and DNA repair in both domains of life [128,129,130]. In addition, cohesins were found to be important for the regulation of gene expression in G1 in eukaryotes, acting through a mechanism of loop extrusion. It became clear recently that a similar mechanism exists in bacteria [131,132,133,134]. Replication in *B. subtilis* was shown to proceed with the help of the loop extrusion mechanism. Upon replication, two large separate hairpins form and condensin rings move in an ATP-dependent manner along contiguous DNA segments, processively enlarging the DNA loops [133] at rates of >50 kilobases per minute [132].

Although more profound exploration is required to broaden the understanding of the role that SMC complexes play in regulating gene expression in bacteria, some SMC–transcription interactions have been demonstrated [135,136]. For example, it was shown that the klesin subunit of SMC is able to interact with at least one two-component sensor kinase whose interaction was confirmed functionally, as well as with other potential kinases and transcription regulators [135]. Head-on transcription was shown to interfere with SMC translocation over the chromosome [136]. A similar effect of transcription on cohesins was recently observed in eukaryotes [137].

The examples and explanations above make it obvious that the details of molecular mechanisms of regulation of bacterial transcription have analogues in the world of eukaryotes.

## 6. Mechanisms of Transcription Activation in Archaea

Current models of eukaryogenesis have undergone significant revision recently [138]. Modern research places the origin of eukaryotes in the archaea clade. The pattern formally corresponds to the two-domain tree of life [139,140]. There is no doubt that eukaryogenesis was a more complex event than previously thought, uniting the genomes of not two, but at least three prokaryotes [138]. Archaea combine the features of bacterial and eukaryotic regulation [55]. However, their mechanisms cannot be directly compared with bacterial ones because the principles of activation are fundamentally different, due to the eukaryotic-like structure of their transcriptional apparatus. In addition, their activation occurs in chromatin, which is fundamentally different from bacteria. Altogether, this makes archaea comparable with eukaryotes rather than with bacteria.

### 6.1. Archean Transcriptional Apparatus: A Combination of Eukaryotes and Bacteria

Archaea are characterized by a simplified version of eukaryote-like general transcription machinery including RNAP and general TFs (GTFs), TBP (TATA-box binding protein), TFB, and TFE (the functional analogs of eukaryotic TFIIB and TFIIE, respectively). Like eubacteria, archaea have one RNAP, while eukaryotes have three. Of these, RNAP most closely resembles the archaeal enzyme. Ten of 13 subunits of RNAP are identical between eukaryotes and archaea, and only 5 are identical to bacterial RNAP [141,142].

Eukaryotes inherited the transcription apparatus and principles of its regulation, including differential transcription regulation by multiple GTFs, TBP, and TFB, and activators/repressors from archaea [55,141,142]. Several archaeal species contain multiple, divergent *tbp* and *tfb* genes, and their multiplicity was hypothesized to have a regulatory role at a higher level, reminiscent of alternative σ factors in bacteria [143]. In turn, activators and repressors encoded by archaeal genomes are closely related to bacterial TFs [55,142,144]. Thus, a little space remains in eukaryotes on the transcription regulation mechanisms specific to them, those that have no analogues in bacteria and archaea. Such mechanisms should apparently be associated primarily with remote communication of DNA regulatory elements scattered in the genomes of multicellular eukaryotes over long distances.

Bacterial RNAP initiates transcription without the involvement of additional factors with the exception of the σ factor, whereas archaeal RNAP requires a minimal set of GTFs, TBP, and TFB. The archaeal core promoter has minimal structural differences from the eukaryotic core promoter [145]. The GTFs of archaea and eukaryotes (TFB/TFIIB, TFE/TFIIE, and MBF1) share no similarity between each other and with bacterial σ factors beyond the presence of distinct DNA binding domains (DBDs). The same situation is observed for gene/operon-specific TFs. Thus, the DBDs might have been independently inherited from a common ancestor in the bacterial and archaeal/eukaryotic lineages. During subsequent evolution, the similarity between archaeal and bacterial regulators might have been established and maintained through multiple horizontal gene transfer events [146].

TFs of archaea are much more similar to bacterial ones than to eukaryotic ones. Approximately 53% of all TFs identified in archaeal genomes have at least one homologue in bacteria, as opposed to 2% having a eukaryotic homologue [147]. However, there is an underrepresentation of ligand-binding domains in the archaeal TFs. This may be compensated for by the formation of different combinations of monomers, like what is observed in eukaryotic transcriptional machinery [146,147]. However, sensing domains are often unique for archaea [144].

### 6.2. Enhancer-Like Mechanisms in Archaea

**Figure 2:**
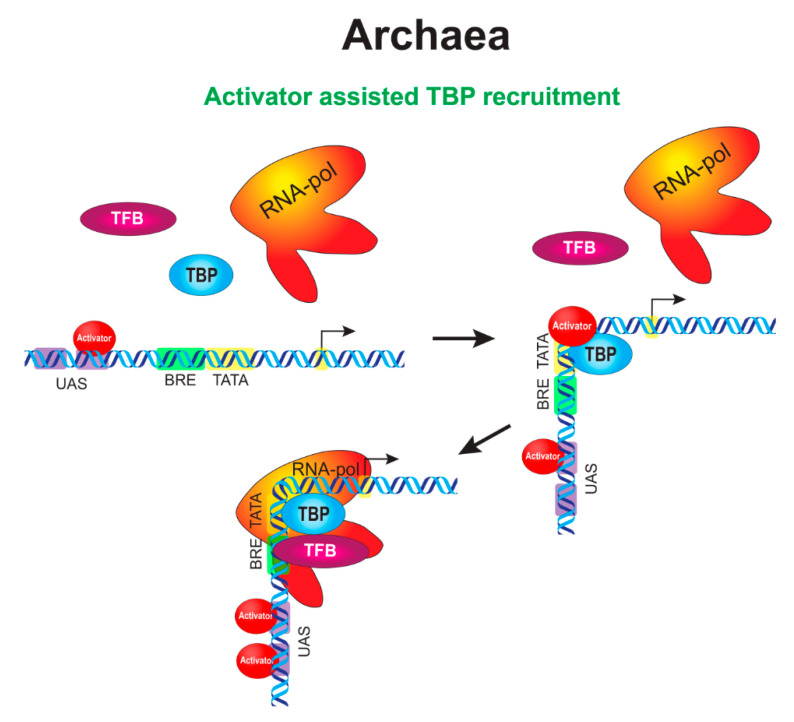
An integrated circuit of the initial stages of transcription activation in archaea. When an incoming signal arrives, a transcriptional activator acquires the ability to bind to its DNA sites in the upstream activating sequence (UAS) region in the promoter of a target gene. Activator binding occurs both in the primary site, which is closest to the promoter, and auxiliary sites, which are distal to the primary site. The appearance of nucleation sites near the core promoter allows the activator to guide TBP to the TATA box (or TFB to the BRE element). This is accompanied by DNA bending in the promoter region, like in the case of activation in bacteria, bringing the regulatory elements of the promoter closer together. The activator may leave the complex when the complex proceeds to the stage of transcriptional bubble or stay in the complex until promoter clearance by RNAP (not shown).

Most archaeal repressors function as in bacteria by abrogating access of one or more components of general transcription machinery to the core promoter [148]. While activation mechanisms are eukaryotic-like and based on the recruitment and stabilization of TBP or TFB to their respective promoter elements [149,150,151,152,153,154]. Some activators are able to interact with both TBP and TFB [155,156] (Figure 2). Moreover, it was shown that one activator can interact with five different TBPs [156]. In contrast to repressors, which can function in combination with any promoter strength, archaeal activators are generally associated with promoters that have larger deviations from the consensus BRE and/or TATA box sequences, as in the case of bacteria [152,157,158].

Activator binding sites in archaea are preferentially located immediately upstream of or partly overlap the BRE element and are often called UASs, as in yeasts [153,159]. At the same time, some of the sites may occur in the distal promoter far from the TSS [160], like in higher eukaryotes. There may be several binding sites in the proximal promoter in the case of archaeal UAS. The site that is closest to the promoter is called the primary site and is critical for activation, while the other upstream sites serve as auxiliary ones (including those located after the TSS). This is similar to the findings in higher eukaryotes, in which the first enhancer in an enhancer chain was shown to be more important. This enhancer forms more stable contacts with the promoter and is important for initiating a transcription bridging of the promoter with other regulatory elements in the locus [161]. The auxiliary sites in archaea are necessary for nucleation and act to increase the concentration of the activator near the primary site and the core promoter. Both wild-type 21-bp spacing (two DNA helical turns) between the primary site and the TATA box and the proper facing of the activator and TBP on the same side of the DNA helix appear to be critical for promoter activation in archaea [153,159].

**Figure 3:**
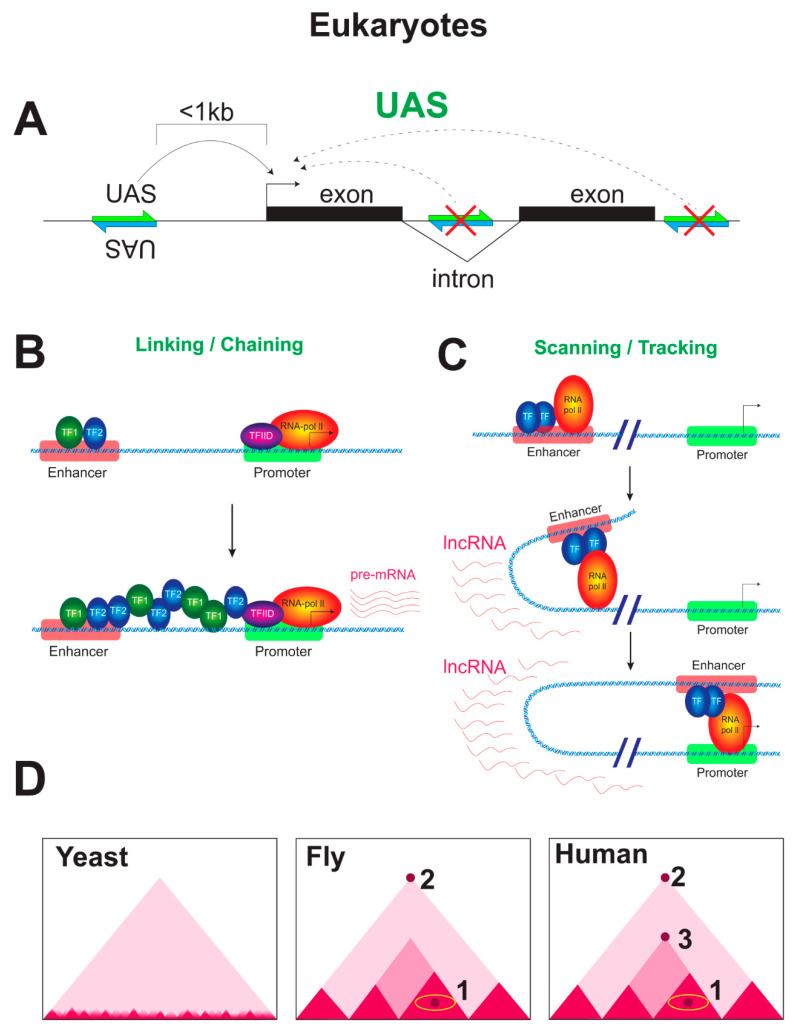
Mechanisms of transcription regulation by enhancers in eukaryotes. (**A**) The regulatory system of yeasts. The UAS works in orientation-independent manner only when placed upstream of the promoter, but not downstream of the gene or in an intron. The yeast UAS is usually close to the promoter (at the distance less than 1 Kb). (**B**) Linking/Chaining mechanism of enhancer–promoter communication. A linking factor oligomerizes from the enhancer to the promoter using some anchored proteins to bridge the distal regulatory elements. This cascade of recruitments starts at the enhancer and proceeds until reaching the core promoter, where the preinitiation complex (PIC) is recruited. Alternatively, oligomerization factor-containing complexes might bind directly to relatively hyperacetylated chromatin in the absence of any anchor protein, possibly, by recognizing a signature of active loci, such as a specific pattern of histone acetylation. (**C**) Scanning/Tracking mechanism of enhancer–promoter communication. An activator binds to the enhancer region and utilizes its activating domain to recruit the other components of the initial transcription complex to the region of the enhancer, the set including basal factors, RNAP, and coactivators. RNAP moves from its initial binding site at the enhancer towards the core promoter, producing non-coding RNAs, which may also represent a platform for the binding of regulatory proteins and coactivators. Although the recruitment of the factors relevant for the initiation of transcription occurs at the enhancer region, transcription is not effectively initiated because the enhancer lacks all motifs necessary for the efficient binding of basic factors that ensure the recruitment of RNAP. At the core promoter, the total set of motifs favorable to preinitiation complex assembly is available and, after the formation of a stable transcriptional complex on the core promoter, the DNA loop between the core promoter and the enhancer is stabilized. The process of RNAP movement towards the core promoter may be accompanied by a wave of unidirectional spreading of histone acetylation through the DNA that separates the enhancer and the promoter (not shown), as well as by histone remodeling in the promoter region. Thus, scanning/tracking can be accompanied by the formation of an acetylated domain between *cis*-regulatory elements, as well as the formation of regions with a reduced density of nucleosomes. (**D**) Genome architecture of eukaryotes (schematic Hi-C maps are shown). Yeasts are devoid of TADs and have only self-associating domains (micro-TADs), which are associated with gene loops, and ~200 Kb TADs, which are associated with DNA replication. In *Drosophila*, the architectural proteins can mediate the interactions of *cis*-regulatory elements inside TADs (indicated with 1). Additionally, flies have long-range looping interactions, which are associated with the function of Polycomb silencing complexes (the long-range looping interactions are indicated with 2). Humans possess all of the mechanisms that mediate enhancer–promoter communication in *Drosophila*, but, unlike flies, mammals additionally have a cohesin-dependent loop extrusion mechanism. The CTCF protein is found at the bases of TADs of different sizes in mammals and is believed to be a master regulator of the chromosomal architecture, while *Drosophila* CTCF acts as an ordinary architectural insulator protein. Cohesin-dependent loop extrusion is an evolutionary acquisition of mammals compared with *Drosophila* (cohesin-CTCF mediated interactions are indicated with 2,3).

Distal sites are often utilized by Ribbon-Helix-Helix (RHH)-family activators [160]. It is unknown currently whether the RHH activators function through large loop formation, as it was described for bacteria in Section 5.4. (Figure 1D), or RHH activators employ a wrapping mechanism and reel up the DNA around themselves. It was shown that activators of the RHH family readily oligomerize into octamers upon crystallization [162,163,164]. The latter variant resembles a linking mechanism observed in eukaryotes (Figure 3B). The necessity of oligomerization for activation was shown for at least some of the RHH proteins [165]. Hence, the following model of wrapping/linking was proposed. The RHH proteins tend to preferentially bind to high-affinity sites located in the distal promoter region. At higher regulator concentrations, RHH proteins begin to oligomerize along DNA towards the core promoter, leading to the occupation of degenerate (low-affinity) binding sites and subsequent activation. In other cases, the process is accompanied by repression when oligomers presumably interfere with preinitiation complex (PIC) assembly [165,166].

One more example of eukaryotic-like regulation involves a transcription activator that has binding sites not only in the proximal promoter, but also in the core promoter. The sequence in the core promoter is located immediately downstream of the TATA element [167], analogous to the BRE^d^ element in RNAP promoters [168].

Some archaeal regulators appear to be of dual nature and exert opposite regulatory effects at the same promoter in a concentration-dependent manner [169,170,171]. These regulators activate transcription at lower concentrations, when not all binding sites are occupied at the promoter and repress the promoter at higher concentrations. This type of regulation presumably involves the formation of a repression loop [148,172]. The formation of a highly dense region in which DNA appears to be wrapped was confirmed by atomic-force microscopy [173].

Thus, Archaea combine the principles of the regulation observed in the other two domains of life, Bacteria and Eukarya. The regulation by multiple TBP and TFB factors is an intrinsic trait of Archaea that has been fully inherited by Eukarya. The regulation in the chromatin is not considered here; however, it is a second common feature between Eukarya and Archaea. The molecular details of these systems are just emerging, and our understanding lags behind the understanding of bacterial systems. It is possible that more types of sophisticated regulation will be found in archaea in the near future.

## 7. Mechanisms of Transcription Activation in Eukaryotes

### 7.1. DNA Topology and Control of Gene Transcription in Eukaryotes

As was mentioned above, the prokaryotic mechanisms resemble or even parallel the mechanisms found in eukaryotes. However, the eukaryotic systems of regulation are generally more sophisticated and include proteins with intricate multidomain structures. Gene regulatory networks are more complex in eukaryotes as a result of several genomic duplications that occurred on the way to vertebrates. The duplications gave origin to paralogs, sometime several. For example, there is a single SMC condensin gene in eubacteria, while multicellular eukaryotes developed several similar complexes with overlapping, but not identical functions. The fixation of a large number of SMC genes in evolution of eukaryotes emphasizes their significance for the function of the eukaryotic genome. Due to huge sizes of higher eukaryotic genomes, some of these complexes, which are known as cohesion complexes, acquired a function in the regulation of gene expression by the loop extrusion mechanism in interphase; their role in enhancer–promoter interactions is now well characterized in eukaryotes [77,80,81].

Like in prokaryotes, the DNA topology has a fundamental control over promoter activity and the ability of TFs to access their target DNA sites in eukaryotes. On the one hand, *cis* regulatory elements dispersed over long distances should be brought together for proper activation or repression. On the other hand, the abundance of DNA-binding proteins subtly sensing the DNA shape begun to open up in eukaryotes. For example, recognition of the DNA shape by proteins and nucleosomes was recently found to involve the A-tracts as short as three base pairs in the DNA minor groove and received the name of indirect readout [174]. Sensing the electrostatic potential in the minor groove is contrasted to the direct readout mechanism that involves the reading of the base sequence in the DNA major groove. Although examples of such a type of regulation are still not abundant in eukaryotes [175,176] and detailed investigation is necessary, these findings partly explain why the binding sites of eukaryotic regulators are typically 10 nt long and are so degenerate [177].

### 7.2. Mechanism of Activator Functioning in Eukaryotes

Before discussing the mechanistic ideas about how enhancers work in eukaryotes, it is necessary to consider the mechanisms whereby eukaryotic activators function because the nature of an activator generally determines the way of enhancer action.

A minimal eukaryotic activator consists of an activating domain (AD) and a DNA-binding domain (DBD). When expressed in eukaryotic cells, such a fusion protein can activate any gene bearing the appropriate DNA binding site nearby. This principle works despite the fact that eukaryotic transcriptional machinery is much more complex than that of bacteria, comprising many proteins in addition to RNAP. A typical eukaryotic activator will recruit whatever factors are required to form the activation complex, and transcription will ensue [178].

The binding of an activator consists in concentration-dependent dynamic interactions, and what is an enhancer to do is simply to increase the concentration of the activator nearby the TSS. Recruitment of an activator merely increases the rate at which the binding reaction occurs. In the absence of an activator, the reaction proceeds spontaneously to some extent, generating a basal level of transcripts. A gene does not stay on once activated unless RNAP is continually recruited by the action of an activator. At abnormally high concentrations of TFs, the specificity can be lost: activators will bind and work where they should not, and enzymes will work on sites that they normally leave unmodified. RNA molecules can also act as recruiters. For example, many regulatory proteins were shown to bind to non-coding RNAs that function to activate their neighbor genes using a *cis*-mediated mechanism [179,180,181].

When contacting enhancers, activators do it by two ways. First, as in archaea, some activators interact directly with basal factors, such as TFIID-TBP or TFIIB and others, recruiting them to the nearest promoter. Second, activators convey their message through coactivators (adapters laying between the activators and basal factors), such as the above mentioned MBF1, the Mediator complex, and histone acetyltransferases (HATs). The spectrum of the recruited factors fully depends on of the AD. For instance, the most powerful AD of the herpes simplex virus VP16 protein recruits almost all basal factors, including several TAFs; coactivators, such as NuA4, p300/CBP, PCAF, and SAGA complexes; the SWI/SNF chromatin remodeling complex; and general cofactors, such as PC4, that are capable of interacting with both ADs and basal factors [182].

Activators make chromatin more permissive for transcription by recruiting HATs either directly, through interaction with a specific HAT subunit, e.g., Tra1p, a shared subunit of SAGA and NuA4 complexes [183], or indirectly, through coactivators other than the Mediator complex, such as, for example, the SRC family of proteins, which recruit HAT (e.g., p300/CBP, PCAF) and histone methyltransferases (HMTs, e.g., MLL) [184]. Chromatin remodeling complexes as well as HATs include special subunits necessary for the interaction with activators [185]. In general, multiple coactivators participate in promoter activation in a gene-specific way [186,187].

The remodeling of the RNAP complex due to direct contact of the activator with RNAP is common in bacteria, but exceptional in eukaryotes. For example, it was initially shown that AD of the HBx protein of hepatitis B virus interacts with the RBP5 subunit, which is present in all three RNAP I, II, III [188]. Later studies demonstrated that a ternary complex of RPB5, HBx, and TFIIB is rather formed [189].

In eukaryotes, a remodeling of RNAP is achieved by introducing a chemical modification in its structure, for example, by phosphorylating its CTD. Activators that can interact with positive transcription elongation factor b (p-TEFb), which consists of CycT and the cyclin-dependent kinase Cdk9), simulate phosphorylation of CTD by p-TEFb at Ser2, which causes the conversion of the RNAP holoenzyme from the initiation complex into an elongation one [190,191].

Phosphorylation of CTD of RNAP by p-TEFb or the CDK7 subunit of THIIH is not the only way to remodel RNAP. Activators can involve external third-party cell kinases for this purpose. For example, it was shown that interactions of VP16 with cell kinases other than THIIH potentiate CTD phosphorylation [192]. Cdc2 is one of these kinases [193]. The mechanism interconnects the cell cycle, signaling, and transcription regulation.

Thus, after binding to an enhancer, an activator presents its activation domain, which either directly or through coactivators interacts with chromatin remodelers and components of basal transcription machinery. The interactions facilitate the assembly of a complete set of GTFs on the promoter and the transition of RNAP to initial RNA synthesizes, which is accompanied by phosphorylation of Ser5 in CTD of RNAP. Subsequent interactions of the activator with p-TEBb and, possibly, CTD transfer transcription into the elongation phase.

### 7.3. Combinatorial Code of Activators

Unlike in prokaryotes, eukaryotic activators do not recognize long and easily recognizable DNA sites, but rather a family of short closely related sequences of up to 10 bp. The circumstance explains why some redundancy is usually observed. To overcome redundancy and to induce a high level of target gene transcription, activators should act in combination with each other, binding multiple regulatory elements in enhancers. In addition, they should act in a proper chromatin context (chromatin context requirements are reviewed in [194]). This combinatorial code is additionally reflected in the fact that, compared with prokaryotic TFs, eukaryotic TFs are often heterodimeric in structure, each of the monomers recognizing a similar, but slightly different sequence. An important principle is that heterodimerization not only yields factors with different affinities for DNA, but also combines activators from different groups, which carry DBDs of different affinities and have ADs of different properties. For example, the thyroid hormone nuclear receptor (TR) can form a TR/TR pair and a pair with the retinoid X-receptor, TR/RXR, which binds DNA more strongly than TR/TR [195,196]. Dimerization of MyoD (see below) with the ubiquitously expressed protein E12 or E47 is required for its maximal DNA-binding activity [197,198]. The c-Myc/Max heterodimer has a higher transactivation ability than the c-Myc/c-Myc homodimer [199,200,201,202].

Eukaryotic regulators often recognize specific DNA sequences in enhancers in a cooperative manner. Cooperativity helps to ensure the specificity of binding and is used to integrate information. Two proteins might exert no effect when present in separate cells but bind (cooperatively) to DNA when present in the same cell. A special case of this principle is the situation where the binding of one activator to one of a few adjacent sites enhances the binding of the same activator to the other sites. The principle is illustrated by the fact that many glucocorticoid response elements (GREs) present in an enhancer stimulate transcription far greater than one GRE does when present alone. The cooperativity of GREs is mediated by its DBD, but not AD [203]. This is one more feature of eukaryotic regulators in addition to their DNA-binding, *trans*-activating, and *trans*-repressing properties.

Sequential cooperativity is another sort of cooperativity and implies interplay of different activators, at least two of them. It is best illustrated by the example where the binding of a first activator (e.g., pioneer TF, see below) to its site enhances/induces the binding of a subsequent activator (of non-pioneer TF nature). Another example is related to the myogenin enhancer, where the MyoD activator initially recognizes its binding site in a proper chromatin context. MyoD utilizes its H/C and helix III domains to interact with an adjacent protein complex containing the homeodomain protein Pbx, which appears to be constitutively bound at this site. Thus, MyoD is necessary for the initiation of chromatin remodeling, but binds second at this site [204].

The principle of cooperativity is additionally expressed at the level of interplay between activators, where different activators recruit different coactivators, enhancing the effect of each other. For example, one activator recruits TFIID, another one recruits TFIIB or HAT, and a third one recruits a chromatin remodeling complex. A cumulative effect is created in this way by the activators, which access their own sites through different mechanisms.

## 8. Current Models of Enhancer Functioning in Eukaryotes

The specificity of an activator is determined solely by the location of the DNA site recognized by its DBD. For example, the yeast transcriptional activator Gal4 can activate essentially any gene when artificially expressed in another eukaryote, provided that the gene has Gal4 binding sites inserted nearby [205] (Figure 3A). However, as mentioned above, both activators and repressors work in eukaryotes even when positioned at any of a wide array of positions on DNA, often at considerable distances from their target genes. The interaction between proteins bound to well-separated DNA sites is accomplished via DNA looping. This kind of «action at a distance» is a rule, especially in eukaryotes with huge genomes (see part 9).

### 8.1. Initial Universal Stages of Enhancer Action

Several models have been presented in the literature to explain how enhancers of eukaryotes function. The key point at which all models converge is the presence of the so-called pioneer TFs (pTFs, or reprogramming factors) that can recognize their target sites in the context of compacted («closed») chromatin, which is covered by nucleosomes and is insensitive to DNase I and other nucleases. This is especially characteristic of developmental genes, which are typically embedded in «closed» chromatin. Thus, activators that potentiate transcription in development are inherently capable of initiating chromatin opening events. However, heterochromatin (carrying the H3K9me2/3 mark) appears to be insensitive to pTFs [206], and chromatin opening is therefore attributed to facultative chromatin created by Polycomb proteins, which regulate developmental genes. pTFs bind to the enhancer DNA to facilitate the subsequent recruitment of other (non-pioneer) activators/repressors, DNA- and chromatin-modifying enzymes, and chromatin remodelers.

One of the best-studied examples of pTFs is provided by the Zelda factor, which acts to establish (but not to maintain) active loop-domains before the midblastula transition in *Drosophila* embryos [44]. Zelda promotes accumulation of the Dorsal activator at the site of an enhancer [207]. Zelda accumulates and forms sub-nuclear dynamic hubs (microenvironments), where its binding events are transient, and facilitates transcriptional activation by accelerating the duration of multiple pre-initiation steps [208]. The exact mechanisms by which such facilitation occurs remain to be determined.

Direct nucleosome- and chromatin-binding studies are useful in order to investigate how exactly the pTFs mechanistically engage chromatin to initiate reprogramming and trans-differentiation of cells [206]. Interesting details were observed for the FoxA proteins, which belong to the large forkhead box (Fox) family (the *Drosophila forkhead* mutation causes defects in head fold involution). Mammalian FoxA opens compacted chromatin in nucleosome arrays assembled with linker histone H1 and containing the albumin gene enhancer in vitro, acting independently of the SWI/SNF chromatin remodeling ATPases, which are frequently required for chromatin opening [209]. This effect of FoxA is mediated by a high-affinity DNA binding site and the C-terminal domain, which binds core histones H3 and H4. FoxA is thought to open chromatin by replacing the linker histone with the use of their wHTH domain, which is highly similar in structure to that of H1 [210,211]. Direct binding to core histones is consistent with the finding that FoxA binds more stably to nucleosome core particles than to free DNA, is not affected by histone acetylation [212], and is facilitated by H3K4me2 [213]. Thus, pTFs translate epigenetic signatures into changes in chromatin structure, thereby helping to establish the lineage-specific transcriptional enhancers and to execute the respective programs.

Another example of how pTFs work is provided by MyoD, a master regulator of muscle differentiation. A cascade of chromatin events was assumed to occur upon myogenin gene activation by MyoD. MyoD is recruited to its target gene enhancers via interaction with the Pbx-Meis complex, which is constitutively bound to the genes prior to activation. p300/CBP acetylates histones H3 and H4 within the promoter region to relax chromatin and then recruits lysine acetylase PCAF. Once tethered to the promoter, PCAF acetylates MyoD to facilitate the trans-activation process [214]. Simultaneously, trans-activation by the AD of MyoD is suppressed by MEK1 kinase (a component of MAPK signaling pathway), which binds to the AD of MyoD [215]. Subsequently, a BRG1-containing SWI/SNF complex is also recruited to the promoter to remodel nucleosomes and to stabilize the DNA binding of MyoD [216]. The order of events may vary depending on the MyoD target genes.

### 8.2. Enhanceosome Complex Assembly

The complex of regulatory proteins on the enhancer is called the enhanceosome. The enhanceosome was crystallized for the interferon-β (IFN-β) enhancer. Transcriptional activation of the IFN-β gene requires that a complex containing the ATF-2/c-Jun, IRF-3/IRF-7, and NFκB activators is assembled on its enhancer. The factors bind to the IFN-β enhancer cooperatively and recruit coactivators to the IFN-β promoter. A crystal structure shows the association of eight proteins with the enhancer [217]. The activators are very close to each other on DNA, and contacts with virtually every nucleotide pair account for the evolutionary invariance of the enhancer sequence. Paucity of local protein–protein contacts suggests that cooperative occupancy of the enhancer comes from both binding-induced changes in DNA conformation and interactions with additional components, such as CBP and probably HMG-group proteins binding the A-T rich minor groove.

The assembly of an enhancesome was studied in vivo with another example. It was found that assembly is hierarchically ordered with the Sox2 protein engaging the target DNA first, followed by binding of Oct4. Sox2 employs a search mode, sliding along open DNA to efficiently locate its targets. Assembly takes about 15 s, including prior three-dimensional diffusion and nonspecific collisions of the factors [218]. The example showed the TFs dynamics and the influence of the epigenome on target search parameters.

### 8.3. Mechanistic Models of Enhancer Functioning

Several mechanical models were proposed to explain the action of enhancers over distance. In a tracking/scanning model [219] (Figure 3C), an enhancer-bound complex, which contains DNA-binding factors (the enhancesome) and coactivators, «tracks» via small steps, or short jumps, or small loops (and possibly scanning) along chromatin until it encounters the cognate promoter, at which a stable looped structure is established [220]. Although small loops are more likely to form than large loops at first glance, this model is doubtful currently, after the discovery of the loop extrusion mechanism functioning in vivo. ncRNAs produced by RNAP are found between the enhancer(s) and promoter (for details, see [194]). The ncRNAs directed towards the promoter may reflect the process whereby RNAP seeks for stable binding to a promoter [221]. Coactivators, such as p300/CBP and ATP-utilizing chromatin remodelers, may modify and remodel the chromatin substrate between the enhancer and promoter and thus may facilitate their communication and the tracking of the enhanceosome complex along the chromatin fiber. Some evidence confirming the mechanism was indeed obtained in vitro. An initially independent assembly of regulatory complexes was observed at the proximal promoter and upstream enhancer regions and was followed by movement of the upstream complex, accompanied by a unidirectional spreading of histone hyperacetylation and remodeling of the nucleosomes situated at the TSS [222].

An alternative model exploits a hypothetical linking/chaining mechanism [223] (Figure 3B), according to which protein–protein oligomerization bridges a distal enhancer and a target promoter. An activator first binds to the enhancer and facilitates the recruitment of a second TF to a site located just downstream of the former. This cascade of recruitments occurs until the core promoter is reached and finally builds a landing platform for general transcription machinery at the TSS [224]. The Chip protein of *Drosophila* was initially identified as a «facilitator» of long-range interactions, and it was assumed that communication between an enhancer and a promoter is mediated by a chain of Chip-containing complexes that are anchored to the chromatin fiber by interactions with homeodomain proteins (HDPs) [225]. Yeast HDPs bind DNA in vivo only in conjunction with other factors [226]. It was shown biochemically and genetically that Chip interacts with LIM-domain proteins [227]. In turn, LIM domains mediate diverse protein–protein interactions. Many HDPs contain the LIM domains. Moreover, mammalian Chip homologs are able to interact with P-Otx, which is a HDP that lacks the LIM domain and is required for synergistic activation of a reporter gene via the bridging of another LIM-domain protein and another HDP and their recruitment to individual promoter proximal sites [228]. Thus, the model of linking/chaining had some kind of biochemical basis. Alternatively, it was assumed that Chip-containing complexes might bind directly to relatively hyperacetylated chromatin in the absence of HDP, possibly by recognizing a feature of active loci, such as a specific histone acetylation pattern. The initiation of a chain of these Chip-containing complexes also takes place on the enhancer in this case [223]. However, studies with Lbd1, a vertebrate ortholog of Chip, suggest that Lbd1 might actually form targeted loops, rather than chains, through homodimerization when bound at enhancers and promoters [229]. At the b-globin locus, the GATA1 activator mediates promoter loops independently of cohesin by interacting with Lbd1 [230].

The targeting looping model is now more or less dominating in the case of multicellular eukaryotes. Even for yeasts, activation with UAS was demonstrated to proceed through the looping mechanism [32].

## 9. Global Genome Organization of Eukaryotes and Enhancer Functioning

The principles of metazoan genome organization were clearly identified in recent years. It was found that the genomes of multicellular eukaryotes are organized in hierarchic layers, which represent structural and functional building blocks of the genome (reviewed in [231]). The layers are (top to bottom): compartments A and B [232], formerly known as euchromatin and heterochromatin; giant CTCF/cohesion-dependent loop domains (insulated neighborhoods) [233,234]; and contact domains [235] or compartmental domains [236]. The latter three are collectively called TADs and subTADs. This area of research, called 3D Genomics, is rapidly developing now, and the terminology used to denote different types of these domains has not yet been fully established. Some attempts in this direction have been made [237]. In this part, we focus on the mechanistic models of enhancer functioning inside TADs.

### 9.1. TAD Level of Enhancer Functioning

TADs and subTADs are the level at which enhancers function. TADs restrict the enhancer action to prevent cross-reactivity between enhancers and promoters from different TADs and, on the other hand, facilitate the interaction between *cis*-regulatory elements located in one TAD [238]. The process of cohesin-mediated CTCF-dependent loop extrusion provides a basis for TADs and giant loop formation, bringing together distant regions of regulatory DNA in vertebrates [77,239]. To bring distal DNA regions to each other, the N terminus of CTCF interacts with the SA2-SCC1 subunits of the cohesion complex in mammals [78].

To monitor the TAD/loop formation biochemically at high resolution, special Hi-C and micro-C methods were developed based on the principle of cross-ligation of DNA fragments after digesting formaldehyde-fixed nuclei with micrococcal nuclease (MNase) or restriction endonuclease [234,240]. Using MNase treatment, loop extrusion was recently studied in mammals at the nucleosomal level at an unprecedented level of resolution. It was found that many of the newly identified loops that bridge regulatory elements and many newly identified peaks are localized along extrusion stripes [241]. Thus, loop extrusion is accompanied by weak loop extrusion pulses, i.e., cohesin complexes stop its progress from time to time.

Profound explanations were recently obtained for how the polarity of CTCF binding and the CTCF protein structure determine the genomic distribution of chromatin loops, bypass of CTCF sites and probably regulatory elements, and overall changes in chromatin organization [242]. Although the general picture is becoming more and more understandable, some difficulties in understanding still persist and direct mechanistic explanations of how loop extrusion proceeds remain to be determined. For instance, loop extrusion was recently demonstrated in vitro for the yeast condensin complex. The process was ATP dependent and occurred at a speed of 1.5 Kb/s, but was found to proceed only in one direction [243]. The ring size of a SMC complex (∼50 nm) exceeds that of a single nucleosome (∼10 nm), and nucleosomes would constitute challenging obstacles for SMC translocation if maintaining constant contact with DNA upon translocation. Thus, additional studies are needed to figure out how exactly loop-extrusion proceeds and how the CTCF complexes stop cohesin.

Although lack of the CTCF protein is embryonic lethal in mammals, *Drosophila* CTCF null mutants (completely lacking both maternal and zygotic CTCFs) survive past the larval stage and die as late pupae with visible defects associated with *Hox* gene misexpression [238]. These genetic data are supported by biochemical observations indicating that a mechanism of CTCF–cohesin-mediated loop extrusion is absent in invertebrates [236]. Collectively, these data suggest that invertebrates can use a more ancient and probably more universal way to mediate the interactions of remote *cis*-regulatory elements. For example, the role might be played by other molecular machines that depend on ATP hydrolysis and are capable of DNA translocation, for example, chromatin remodeling complexes [244].

While lacking the loop extrusion mechanism, *Drosophila* is not devoid of ways to bring remote regions of the genome close together. Long-range looping interactions were initially not found in *Drosophila* in early genome-wide studies using the Hi-C method because of the technical imperfections of the method at early stages of its development [245,246]. Later, a small number of *Drosophila* domains with apical interaction hotspots were identified in early embryos [247] and Kc embryonic cells [42]. Although CTCF binding sites do overlap cohesin ChIP-seq peaks [248], they do not exhibit a CTCF motif orientation bias at domain borders [244] and do not anchor Hi-C peaks [42,249]. Some of these contacts obviously represent rare selected interactions between distant regulatory elements, and some others are contacts between PREs and relate to interactions between Polycomb silencing complexes. PRC2-dependent loop formation was recently confirmed in vivo in embryos and S2 embryonic cells and in vitro in *Drosophila* [88], as well as in vitro using atomic force microscopy in liquid for human PRC2 [90]. PRC1-dependent loop formation independent of CTCF was recently shown in mouse embryonic stem cells [89].

Unicellular eukaryotes (yeasts) were shown to possess two types of TADs, ~200-kbp TADs, which regulate replication timing so that origins within a domain fire synchronously [250], and self-associating domains (micro-TADs), which typically encompass one to five genes [240]. None of these TAD types resembles the types habitual for metazoa. Later research identified the components of yeast basic transcriptional machinery—Mediator, Ssu72, and Rtt109 H3K56 acetyltransferase—that mediate chromatin organization into self-associating domains through the mechanism of gene loop formation, when the beginning and end of a gene are brought together. In addition, it was shown that the RSC chromatin remodeling complex, which normally occurs at promoters of highly transcribed genes, participates in organizing the boundaries of self-associating domains [240].

Thus, the examples above provide evidence that the way the genome is organized may change the principles of regulation by enhancers (Figure 3D). Vertebrates possess a long-range looping interaction system based on the cohesin-dependent loop extrusion mechanism and obviously the mechanisms that provide for enhancer–promoter contacts within TADs. Invertebrates do not have a perfect system for gene regulation by the cohesin complex, despite its obvious requirement for cell division. Therefore, gene regulation in invertebrates, like in yeasts, should obviously rely on the mechanisms that work inside TADs.

### 9.2. Principles of Activation by Enhancers Inside TADs

In multicellular eukaryotes, the principles of activation inside TADs might be exactly the same for both invertebrates and vertebrates because the evolutionary distance does not allow any fundamental molecular difference to develop. Instead of CTCF, which occupies TAD borders in mammals, a lot of architectural proteins were detected on the borders between TADs in *Drosophila* from the very beginning [245,246]. Some of them were assumed to play a role in establishing and maintaining the enhancer–promoter interactions, by bridging DNA sites in the enhancer and promoter or acting thorough protein–protein interactions. The concept received some confirmation. First, two specific types of enhancers, that is, enhancers of housekeeping and developmental genes, were found to exist in the *Drosophila* genome. Second, each class of enhancers was demonstrated to have its own preferences regarding the architectural protein composition [249]. A functional significance was demonstrated for certain zinc-finger architectural proteins, which were found to support the long-range interactions between the enhancer and the promoter [35]. Based on their large number, it cannot be ruled out that some of the architectural proteins may act as coactivators of transcription when placed or attached to chromatin.

Thus, in *Drosophila*, the enhancer–promoter specificity is achieved within a TAD with the use of a compatibility code between enhancers and classes of core promoters [251,252]; special enhancer-bound and promoter-bound architectural proteins that interact with each other [253]; and tethering elements, which are specific parts of proximal promoters or enhancers and provide for enhancer–promoter communication over great distances (reviewed in [254]).

In vertebrates, the regulatory system based on architectural proteins is not as obvious as in *Drosophila,* possibly because vertebrates possess the cohesin/CTCF-mediated loop extrusion system. However, some examples of architectural proteins were discovered. LBD1 (Chip in flies) and YY1 [255], studied far better, may execute analogous architectural functions in mammals, bringing enhancers and promoters together. As it should be expected from a mammalian regulator, YY1 appears to be multifunctional; acts through homodimerization both as a repressor and as an activator of gene expression [256], depending on the context, as we saw for prokaryotes above; and may act in cooperation with other factors [257]. Widespread loss of H3K27 acetylation was observed on YY1-bound enhancers, underscoring the crucial role of YY1 in enhancer regulation [258]. YY1 binds to both gene regulatory elements and their associated RNAs, indicating more complex interplay than that observed for bacterial activators [181]. YY1 may act as an auxiliary protein of Polycomb repressive complexes [259]. In addition, acting as an activator, YY1 recruits the INO80 remodeling complex to YY1-activated genes, where INO80 functions as a YY1 essential coactivator. Binding of YY1 to its DNA sites in target genes requires INO80, suggesting that YY1 uses the INO80 complex not only to activate transcription, but also to gain access to target promoters [260].

Thus, in the near future, the rapidly developing field of studying loop extrusion in mammals will move from accumulating general ideas of how it works to mechanistic justifications. Some examples of such a transition are already available [243], but still not numerous. At the same time, studies in invertebrates will solve the problem of understanding the role of architectural/insulator proteins and their function in the regulation of transcription. The problem still remains unsolved in spite of more than 30 years of relevant research.

## 10. Final Remarks

### 10.1. Parallels of Regulatory Evolution

The origin of the mechanisms of transcriptional regulation lies in the world of prokaryotes, and we will never know what the first mechanisms of regulation were. However, using the existing diversity of living beings, we can reconstruct the evolution of regulatory mechanisms, understand its laws, and highlight the fundamental differences. The first thing worth paying attention to is the principle of initiation of transcription by RNAP. Recent findings indicate a common origin of initiation factors: the bacterial sigma subunit of prokaryotes and TFB and TFIIB of archaea and eukaryotes, respectively [261]. The discovery of initiating TFIIB factors for RNAP I emphasizes that not a single RNAP of cellular organisms is capable of initiating transcription independently, testifying again to the evolutionary conservation of transcription initiation origin [262].

Regulation by multiple initiation factors is another trait used by all cellular organisms. In bacteria, such factors belong to the family of sigma subunits, while multiple TBPs and TFBs are used in archaea. This feature was inherited by eukaryotes from archaea. For example, in Metazoa, transcription is regulated by TBP and its TBP homologs, TBP-related factors [263,264].

One more general principle that has been preserved in evolution of all groups is compensation for weak motifs of the core promoter by activators. Activators recruit sigma-initiating subunit of bacteria and functional analogues of sigma, TBP+TFB, in archaea. In different types of eukaryotes, activators perform the same job and additionally recruit various auxiliary complexes that facilitate operations with nucleosomes.

### 10.2. Gradual Increase of Regulatory Complexity in Evolution of Eukaryotes

Although the eukaryotic system of gene expression regulation is the most sophisticated, eukaryotes use the same principle of recruitment of initiating basal factors to the promoter that they inherited from Archaea. In archaea, activators bring the TBP and TFB to a promoter and facilitate their interaction with RNAP. Such a functionality of archaeal activators resembles one-component coactivator activity of eukaryotes and appears to be very stable in evolution, being maintained from unicellular eukaryotes to invertebrates and finally to vertebrates. For example, the MBF1 factor bridges TBP and the GCN4 activator in yeasts [157] and nuclear receptor FTZ-F1 and TBP in *Drosophila* [158], while its repertoire expands in vertebrates to two activators, SF-1, which is a mammalian counterpart of FTZ-F1 and ATF1 [159,160]. Thus, the principle of recruitment of TBP and TFB by transcription activators, which was developed by prokaryotes about 4 billion years ago, was successfully fixed in evolution and transformed into a coactivator system in eukaryotes.

At the same time, gradual complication of organisms certainly affected the transcriptional regulation and eukaryotes have already acquired one-component heterodimeric coactivators. In addition, eukaryotes acquired multi-component coactivator complexes, such as Mediator, to integrate and transmit the signals from multiple TFs to a few factors of basal transcription machinery; certain other complexes, such as chromatin remodeling complexes, to operate with nucleosome-imbedded DNA; and histone-modifying complexes, to facilitate these operations.

Although examples where the DNA topology affects the activity of a promoter were described in bacteria, activation of transcription in this domain was always accompanied by direct interactions of activators with bacterial RNAP and/or the sigma-initiating subunit. Unlike in prokaryotes, eukaryotic activators convey their message by contacting RNAP through the basal factors, coactivators and the Mediator complex, which surround RNAP, but never interact with RNAP directly.

### 10.3. Perspectives and Problems to be Solved

Despite the advances made in recent decades in studying the regulatory mechanisms in eukaryotes and other organisms, including genome-wide studies, several evolutionary problems of molecular biology are still not resolved and, apparently, will not be resolved in the nearest future. For instance, although NGS technologies made it possible to conduct metagenomics analysis of populations of microorganisms, and representatives of a new archaeal group—direct evolutionary predecessors of eukaryotes—were identified and then cultivated in the past 5 years, accumulating knowledge is still the main feature of the research process and findings of the missing transition links between archaea and eukaryotes do not allow us to obtain comprehensive answers about evolution of specific molecular regulatory mechanisms.

For example, it is still an enigma when and why archaeal activators functionally turned into coactivators of eukaryotes. This can be partially explained by the increase in the number of regulators in Eukarya, where some of the regulators assumed the functions of coactivators by creating an adapter link between the remaining activators and basal factors. Alternatively, it may be explained by multiple gene and whole genome duplications, which occurred in evolution of unicellular eukaryotes and provided greater evolutionary flexibility to regularity mechanisms. This situation is clearly evident in the yeast genome, which has more than several hundreds of duplicated genes and where duplications were accompanied by asynchronous paralog differentiation and regulatory neofunctionalization [265,266,267].

One more enigma is the origin of the Mediator complex. Although several attempts were made to come closer to understanding this problem [268,269], the question of how such a large regulatory complex arose in evolution of eukaryotes remains a mystery. These studies identified a minimal set of core subunits, revealed elongation of the subunits in metazoans and plants, and showed the differences in distribution of intrinsically disordered regions in different kingdoms. It was also shown that none of the subunits is specific to metazoans, supporting a very ancient eukaryotic origin of this large complex.

## 11. Conclusions

Here we described diversity and the gradual increase in complexity during evolution of the mechanisms underlying transcriptional control by enhancers and enhancer-like regulatory elements (eREs) in different organisms (Table 1). A direct contact of the eRE and promoter is a common feature of activated transcription in all taxa. While the contact is due to the close proximity of the eRE to the promoter in DNA sequence in bacteria, specific mechanisms evolved in higher organisms to bring together the elements that are far apart. In general, bacterial transcription is governed by one or few TFs forming direct contacts with RNAP. In addition to TFs themselves, a local molecular environment (the DNA topology and packaging DNA-binding proteins) has an effect on transcription output. Archaea and unicellular eukaryotes are characterized by having evolved a specific layer of protein factors to mediate the TF–RNAP interaction; the layer consists of multiple classes of GTFs and coactivators in eukaryotes. Moreover, eREs become more separable from promoters in the genome. Epigenetic regulation of transcription developed in eukaryotes [270]. Finally, transcription in Metazoa is characterized by extensive control via multiple eREs, each occupied by multiple TFs. Enhancers become dispersed throughout the genome and diversified in function. The separation of enhancers and promoters by long distance and an increase in the abundance of enhancers are accompanied by the appearance of special mechanisms that ensure the convergence of regulatory elements in space. In general, the DNA architecture became more and more important in transcription regulation: other genomic elements, architectural proteins, and higher levels of DNA packaging became actively involved in this process during evolution [271]. However, although the mechanisms of transcriptional control drastically differ between Metazoa and Bacteria, it is still possible to observe the original prototypes of complex eukaryotic mechanisms in Bacteria. Our current understanding of these mechanisms is far from complete [194]. Further studies are necessary because comprehensive knowledge of the function of these genomic elements is highly important for multiple applications in biotechnological and medical fields [272,273].

## Figures and Tables

**Table 1:** Features of transcription driven by enhancers and enhancer-like regulatory elements (eREs) in different taxa.

Features of Regulated Transcription	Prokaryotes		Eukaryotes		
	Eubacteria	Archaea	Yeasts	Drosophila	Human
Proximity of eRE to promoter in space	+	+	+	+	+
Distal location of eRE relative to promoter in DNA sequence	−	−	±	+	+
Control of one gene by multiple eREs	−	−	−	+	+
Hierarchy (classes) of enhancers	−	−	−	+	+
Participation of other genomic elements	−	−	−	+	+
Direct TF–RNAP interaction	+	+	−	−	−
Control of one gene by multiple TFs	−	−	+	+	+
Cooperative action of multiple TFs on one eRE	−	−	±	+	+
Several classes of GTFs	−	+	+	+	+
Participation of coactivators	−	−	+	+	+
Regulation via local DNA topology	±	±	+	+	+
Regulation via histone-like proteins	±	±	+	+	+
Regulation via DNA macrodomain packaging	−	−	+	+	+
Regulation by architectural proteins	−	−	−	±	±
Regulation via chromatin loop extrusion	−	−	−	−	+

–—absent or rare, ±—occasional, +—common.
